# Targeting Autophagy Facilitates T Lymphocyte Migration by Inducing the Expression of CXCL10 in Gastric Cancer Cell Lines

**DOI:** 10.3389/fonc.2020.00886

**Published:** 2020-06-02

**Authors:** Qingyuan Meng, Yihong Zhang, Liangbiao George Hu

**Affiliations:** Department of Comparative Biology and Safety Science, Amgen Biopharmaceutical R&D (Shanghai) Co., Ltd, Shanghai, China

**Keywords:** autophagy, CXCL10, gastric cancer cell lines, JNK, T lymphocyte migration, *in vitro*

## Abstract

Autophagy is a type of cellular catabolic degradation process that occurs in response to nutrient starvation or metabolic stress, and is a valuable resource for highly proliferating cancer cells. Autophagy also facilitates the resistance of cancer cells to antitumor therapies. However, the involvement of autophagy in regulating CXCL10 expression in gastric cancer (GC) cells and T lymphocyte migration remains unclear. In this study, we aimed to investigate the effect of autophagy inhibition on CXCL10 expression and T lymphocyte infiltration in GC and elucidate the underlying mechanism. Analysis of public databases revealed a positive correlation between CXCL10 expression and both prognosis of patients with GC and the expression profile of T lymphocyte markers in the GCs. Chemotaxis and spheroid infiltration assays revealed that CXCL10 induced T lymphocyte migration and infiltration into GC spheroids, an *in vitro* three-dimensional cell culture model. In addition, *in vitro* autophagy inhibition in GC cells increased CXCL10 expression under both normal and hypoxic culture conditions. Further investigation on the underlying mechanism showed that *in vitro* autophagy inhibition suppressed the JNK signaling pathway and further enhanced CXCL10 expression in GC cells. Collectively, our results provide novel insights for understanding the role of autophagy in regulation of intra-tumor immunity.

## Introduction

A correlation between the presence of tumor-infiltrating lymphocytes (TILs) and overall patient survival has been reported in several tumor types (1–4) and the fundamental roles of TILs in tumor immunity have been investigated intensively (5–10). Therefore, immunomodulation using immune check-point inhibitors, one of the most rapidly growing cancer drug classes, is currently being explored as a cancer therapeutic approach. Some immune check-point blockade therapies, such as those involving monoclonal antibodies targeting cytotoxic T lymphocyte associated protein 4 (CTLA-4), programmed cell death protein 1 (PD-1), and PD-1 ligand (PD-L1), resulted in T lymphocyte-mediated tumor regression in various malignancies (11–17), including gastric carcinoma (18).

Gastric cancer (GC) is the fifth most common malignancy diagnosed worldwide, with 952,000 estimated new cases and 723,000 GC related-deaths in 2012 (19). Although immune check-point inhibitors have shown promising results for GC treatment, the objective response rates remain low (18, 20). Thus, the effectiveness of this immunomodulatory strategy depends not only on the unleashing of pre-existing immunity but also on the presence of a baseline immune response (21). In fact, intra-tumor T lymphocyte recruitment is one of the potential rate-limiting steps in immunotherapy; therefore, many investigators have focused on the role of intra-tumoral chemokines in TIL recruitment into the tumor (22, 23).

It is well-known that T lymphocyte infiltration into the tumor is always insufficient when the chemokine receptors expressed on T lymphocytes do not match to the tumor-secreted chemokines (24). CXCR3, a predominant chemokine receptor expressed on TILs, is expressed in several solid tumors, including melanoma (25), colorectal cancer (26), and breast cancer (27). Moreover, TILs in lymphocyte-rich GCs predominantly express CXCR3 (28). Among the CXCR3 ligands, CXCL10 was reported to be associated with T lymphocyte infiltration into tumors. For example, CXCL10 expression was associated with T lymphocyte recruitment in melanoma metastases (25). In addition, intra-tumor induction of CXCL10 enhanced the infiltration of CXCR3+ cytotoxic T lymphocytes, thereby improving the antitumor effect of other therapies in some rodent solid tumor models (29, 30). However, the association between CXCL10 expression and T lymphocyte infiltration in GC remains poorly understood.

In recent years, autophagy in GC pathogenesis has been explored extensively, and autophagy inhibition is being considered as a potential strategy for GC treatment (31). Autophagy is critical for the digestion of intracellular contents and generation of energy to control cellular homeostasis (32). Autophagy was reported to play a pivotal role in GC cell survival and enhance tumor cell resistance to antitumor therapies (31). Therefore, autophagy inhibition may alter this tumor protective mechanism and potentiate anticancer drug-induced cell death in GC. In fact, an autophagy inhibitor chloroquine (CQ) was reported to improve the chemosensitivity of GC cells to platinum-based antitumor drugs (33, 34). Li et al. demonstrated that treatment with 3-MA, an alternative autophagy inhibitor, enhanced the curcumin-induced antitumor effect (35). Interestingly, a recent study showed that autophagy inhibition could induce CCL5 expression in melanoma cells, resulting in tumor regression facilitated by NK cell migration into the tumor bed (36).

In this study, we investigated the effect of autophagy inhibition on CXCL10 expression in GC cells and T lymphocyte migration toward GC cells. We also attempted to elucidate the mechanism underlying the observed effects of autophagy inhibition on CXCL10 expression in GC cells.

## Materials and Methods

### Public Dataset Mining

Kaplan Meier-plotter (http://kmplot.com/analysis/) is an online database that enables evaluation of the effect of over 54,000 genes on survival in several cancer types, including GC, breast cancer, ovarian cancer, and lung cancer (37). This database was used to obtain prognostic information on CXCL10. Survival information and gene expression data were from Gene Expression Omnibus (GEO), European Genome-phenome Archive (EGA), and The Cancer Genome Atlas (TCGA) database.

Gene Expression Profiling Interactive Analysis (GEPIA; http://gepia.cancer-pku.cn/index.html) is a customizable online tool developed by Zhang lab of Peking University to analyze gene expression data in both tumor and normal tissues on the basis of TCGA and Genotype-Tissue Expression (GTEx) data (38). GEPIA was used for correlation analysis and for investigating the expression levels of autophagy-related genes (ATGs) between GCs and the normal tissues.

### Cell Lines and Reagents

Human GC cell lines AGS, NCI-N87, BGC-823, HGC-27, KATO III, SGC-7901, SNU-1, SNU-5, and SNU-16 were purchased from American Type Culture Collection (ATCC). AGS, BGC-823, HGC-27, KATO III, and SNU-5 cells were cultured in DMEM-GlutaMAX medium (Life Technologies) supplemented with 10% fetal bovine serum (FBS; Life Technologies), penicillin (100 U/ml), and streptomycin (100 μg/ml; Life technologies). NCI-N87, SGC-7901, SNU-1, and SNU-16 cells were cultured in RPMI 1640-GlutaMAX medium (Life Technologies) supplemented with 10% FBS, penicillin (100 U/ml), and streptomycin (100 μg/ml). All the cells were maintained in a 5% CO_2_ humidified atmosphere at 37°C. The ATG5 and ATG7 siRNAs were purchased from Life Technologies. CQ, cobalt chloride (CoCl_2_) and Sp600125 were purchased from Sigma. Anisomycin was purchased from Cell Signaling Technology. Recombinant CXCL10 protein, CXCL10 antibody and mouse IgG1 isotype control were purchased from R&D systems. The plasmid pIREShyg3 was purchased from GenScript and the coding sequence (CDS) of CXCL10 gene was cloned in pIREShyg3 using Nhel / BamHI to obtain the pIREShyg3-CXCL10 plasmid.

### Cell Sorting and Activation of CD3+ T lymphocytes

CD3+ T lymphocytes were isolated from cryopreserved human peripheral blood mononuclear cells (PBMCs; StemExpress) using MACS microbeads (Miltenyi Biotec). After separation, T lymphocytes were stimulated using CD3/CD28 Dynabeads (Life Technologies) for 2 days, as described previously, and re-cultured without any external stimuli for another 2 days to induce the expression of CXCR3 (39). The primed T lymphocytes were used in the chemotaxis and spheroid infiltration assays.

### Flow Cytometry Analysis

Cells were incubated with saturating amounts of various fluorescent-labeled antibody mix composed of PerCP-Cy5.5 labeled mouse anti-CD45 (Clone HI30, IgG1; BD Biosciences), PE labeled mouse anti-CD3 (Clone OKT3, IgG2a; Thermo Fisher Scientific), FITC labeled mouse anti-CXCR3 (Clone G025H7, IgG1; BioLegend) antibodies, and co-stained with Zombie Aqua^TM^ dye (BioLegend). Isotype and fluorochrome-matched mAbs were used for control staining. Stained cells were evaluated using the BD LSRFortessa X-200 flow cytometer (BD Biosciences), and the data were analyzed using FlowJo software (Tree Star).

### Chemotaxis Assay

The chemotaxis assay was performed in CytoSelect^TM^ 24-well cell migration assay kit (5 μm pore size; Cell Biolabs) per the manufacturer's instructions (**Figure 2A**). Briefly, the primed T lymphocytes were prepared at density of 3 × 10^6^ cells/ml in serum-free RPMI 1640 medium containing 0.5% bovine serum albumin (BSA), 2 mM MgCl_2_, and 2 mM CaCl_2_. For each well, the cells were placed in upper chamber (3 × 10^5^ cells/100 μl) and the medium was loaded in the lower chamber. The plate was then incubated in a 37°C cell culture incubator for 5 h. The migrated cells were dissociated from the membrane, lysed, and detected using the patented CyQUANT® GR Dye (Life Technologies).

### Tumor Spheroids and Spheroid Infiltration Assay

NCI-N87 spheroids were established using 96-well EZSPHERE SP micro-plates (Nacalai Tesque). The culture plate has a concave and ultra-low attachment bottom surface so that the cells adhere to each other, but not with the bottom surface of the plate. Therefore, the cells did not spread out on plastic, but formed spheroids. Here, the NCI-N87 cells were transfected with the pIREShyg3-CXCL10 plasmid; 1 day later, 8 × 10^4^ CXCL10-transfected NCI-N87 cells were seeded with 200 μl medium in each well. The spheroids were formed 4 days after seeding. Then, 8 × 10^5^ primed T lymphocytes were added into each well and incubated overnight (**Figure 2C**). The spheroids were then washed three times with PBS to remove the loosely attached T lymphocytes, fixed in 4% paraformaldehyde for 2 h, and embedded into paraffin for immunohistochemistry analysis.

### Immunohistochemistry

Paraffin blocks were sectioned using a microtome to obtain 4 μm thick sections for immunostaining. The paraffin sections were dewaxed in xylene and hydrated in decreasing concentrations of ethanol. Sections were then incubate in 1 × DIVA Decloaker antigen retrieval solution (Biocare Medical) at 110°C for 15 min using the decloaking chamber (Biocare Medical). Following antigen retrieval, sections were incubated in peroxidazed 1 solution (Biocare Medical) at room temperature for 5 min to quench endogenous peroxidase activity. After blocked with background sniper at room temperature for 10 min, sections were incubated with a monoclonal rabbit anti-human CD3 antibody (0.3 μg/ml; Biocare Medical) in Dako REAL antibody diluent (Dako) at room temperature for 1 h. Sections were subsequently incubated with HRP-labeled goat anti-rabbit IgG polymer (Dako) at room temperature for 30 min. Finally, sections were exposed to liquid DAB+ substrate chromogen system (Dako) at room temperature for 5 min and counterstaining was performed using Gill's hematoxylin (Sigma).

### Quantitative RT-PCR

Total RNA was extracted from the GC cell lines using RNeasy Plus Mini Kits (QIAGEN). Quantitative RT-PCR and data analysis were performed as described in our previous work (40, 41). Briefly, the SuperScript™ IV First-Strand Synthesis System (Life Technologies) was used to synthesize cDNA. PCR was performed and quantified using Power SYBR Green PCR Master Mix (Life Technologies). Primers used in the real-time quantitative PCR were as follows: CXCL10 (accession no. NM_001565), sense primer 5′- AAAAGAAGGGTGAGAAGAG-3′ and antisense primer 5′- AAGAACAATTATGGCTTGAC-3′; ATG5 (accession no. NM_004849), sense primer 5′-GCAACTCTGGATGGGATTGC-3′ and antisense primer 5′-AGGTCTTTCAGTCGTTGTCTGAT-3′; ATG7 (accession no. NM_ 006395), sense primer 5′-CATGGTGCTGGTTTCCTTGC-3′ and antisense primer 5′- GCTACTCCATCTGTGGGCTG-3′; GAPDH (accession no. NM_002046), sense primer 5′- CGGATTTGGTCGTATTGGG-3′ and antisense primer 5′- CTGGAAGATGGTGATGGGAT-3′.

The relative target gene mRNA level was calculated using the ΔCt method. The threshold cycle (Ct) values of the target gene mRNAs were initially normalized to the Ct values of the internal control GAPDH in the same samples: ΔCt = Ct (the target gene) – Ct (GAPDH). These values were further normalized to the control group: ΔΔCt = ΔCt (sample group) – ΔCt (control group). The fold change was then determined (2^−ΔΔ*Ct*^). The relative target gene mRNA level represents an average fold calculated from separate experiments. PCR was performed at least three times, and similar results were observed.

### Luminex Assay

The protein level of CXCL10 in the cell culture supernatant was assessed using the human Magnetic Luminex Assay (R&D Systems), which was performed per the manufacturer's instructions. Briefly, all the samples and standards were first mixed with the CXCL10 antibody coated magnetic microparticles and incubated for 2 h at room temperature on a horizontal orbital microplate shaker set at 800 rpm. After washing the microparticles, biotinylated detector antibodies were added and incubated for 1 h at room temperature on the shaker set at 800 rpm. Following a wash to remove any unbound biotinylated detector antibody, streptavidin-phycoerythrin conjugates were added and incubated for 30 min at room temperature on the shaker set at 800 rpm. Finally, the protein level of CXCL10 in the cell culture supernatant was analyzed using the Bio-Plex^TM^ 200 system (Bio-Rad).

### Western Blot

Cell lysis, protein extraction, and western blot analyses were performed as described in our previous work (40). Proteins were dissolved in a lysis buffer and separated using SDS/PAGE for western blot analyses. Primary antibodies included rabbit anti-Phospho-SAPK/JNK (Thr183/Tyr185), anti-SAPK/JNK, anti-Phospho-c-Jun (Ser73), anti-c-Jun, anti-ATG5, anti-LC3B and anti-GAPDH (Cell Signaling Technology). Secondary antibody was HRP-conjugated anti-rabbit IgGs (Life Technologies). The densitometric analyses of western blotting images were performed using ImageJ software (National Institutes of Health).

### Cell Viability Assay

Cell counting kit-8(CCK-8, Dojindo) was used to evaluate cell viability based on the dehydrogenase activity. AGS cell suspensions were first dispensed in a 96-well plate (1 × 10^4^ in 100 μL/well) and cultured in DMEM with 10% FBS at 37°C for 24 h, and then were treated with vehicle, 10 and 20 μM CQ, respectively. After incubation for 0, 1, 2, and 3 days, 10 μl CCK-8 solution was added to each well and the plate was incubated at 37°C for 1 h. Finally, the absorbance at 450 nm was measured by using a SpectraMax M5 microplate reader (Molecular Devices).

### Statistical Analysis

Data represent mean ± SE. Experimental data were subjected to statistical analyses using one-way ANOVA followed by Tukey *post-hoc* test or student's *t*-test with a significance level of *P* < 0.05.

## Results

### CXCL10 Expression in GC Was Positively Correlated With Survival and Expression Profiles of Intra-tumor T lymphocyte Markers

**Figure 1:**
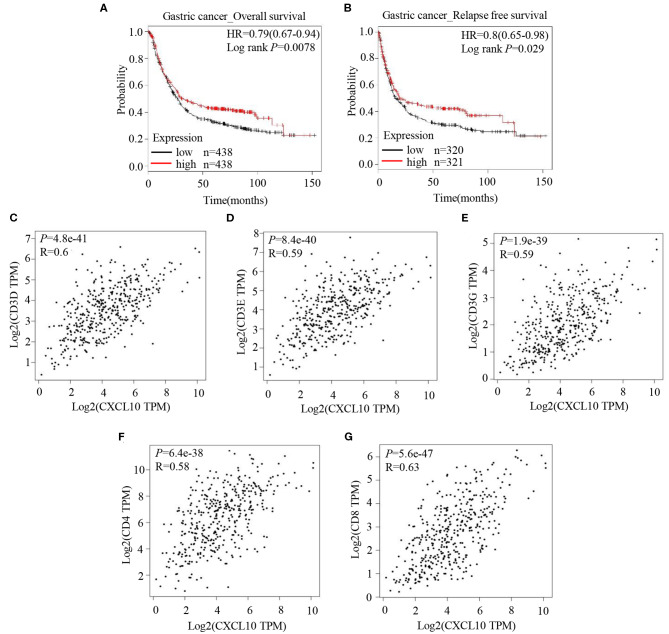
CXCL10 expression is positively correlated with survival and expression of T lymphocyte markers in patients with GC. **(A)** Kaplan-Meier analysis of overall survival in GC patients with high CXCL10 expression and low CXCL10 expression (*P* = 0.0078, *n* = 438). **(B)** Kaplan-Meier analysis of relapse free survival in GC patients with high CXCL10 expression and low CXCL10 expression (*P* = 0.029, *n* = 320 and 321, respectively). **(C–G)** Scatter plots showing the correlation of CXCL10 with CD3D **(C)**, CD3E **(D)**, CD3G **(E)**, CD4 **(F)**, and CD8 **(G)** (Spearman's correlation test).

Analysis of the prognostic information on CXCL10 in cancers (http://kmplot.com/analysis/) revealed a positive correlation of CXCL10 expression with both overall survival (Figure 1A, HR 0.79 [0.67–0.94], logrank *P* = 0.0078) and relapse free survival (Figure 1B, HR 0.8 [0.65–0.98], logrank *P* = 0.029) in patients with GC, but not in patients with breast cancer (Figures S1A,D), lung cancer (Figures S1B,E), or ovarian cancer (Figures S1C,F). In addition, correlation analysis in GEPIA showed strong positive correlation between CXCL10 expression and several T lymphocyte markers such as CD3D (Figure 1C, *P* = 4.8e−41, *R* = 0.6), CD3E (Figure 1D, *P* = 8.4e−40, *R* = 0.59), CD3G (Figure 1E, *P* = 1.9e−39, *R* = 0.59), CD4 (Figure 1F, *P* = 6.4e−38, *R* = 0.58), and CD8 (Figure 1G, *P* = 5.6e−47, *R* = 0.63). These results suggested that the CXCL10 expression in GC might be positively correlated with intra-tumor T lymphocyte infiltration.

### CXCL10 Recruited T lymphocytes in the Chemotaxis and GC Spheroid Infiltration Assay

**Figure 2:**
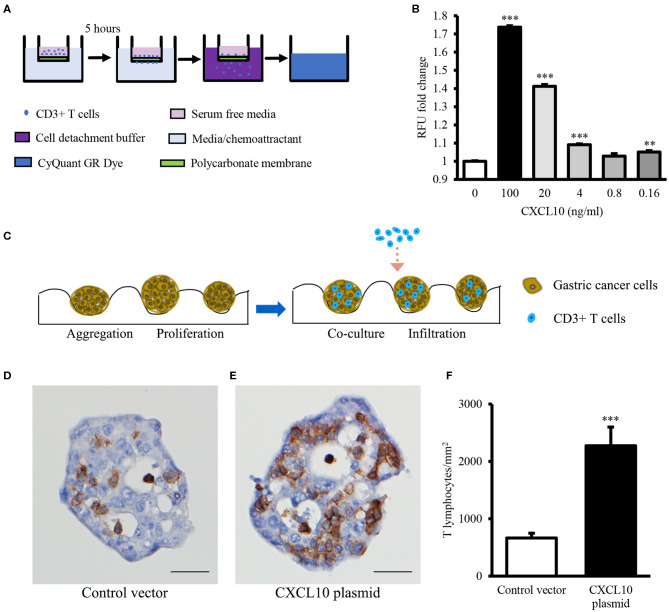
CXCL10 recruits T lymphocytes in chemotaxis assay and GC spheroid infiltration assay. **(A)** Schematic representation of chemotaxis assay for T lymphocyte migration through the polycarbonate membrane toward different concentrations of recombinant CXCL10 protein. **(B)** Statistic analysis of fold change of migrated T lymphocytes. **(C)** Schematic representation of T lymphocyte infiltration into NCI-N87 spheroids. **(D,E)** Representative images of CD3 immunohistochemistry staining in NCI-N87 spheroids transfected with control vector **(D)** or CXCL10 plasmid **(E)**. **(F)** Histogram indicating the density of T lymphocytes in NCI-N87 spheroids. **P < 0.01, ***P < 0.001. Data represent mean ± SE. Scale bar: 25 μm.

Binding specificities of chemokines to their specific receptors are well-defined (42), and high expression of CXCR3 (the receptor of CXCL10) on effector T lymphocytes has been reported (43). Therefore, to confirm whether CXCL10 induces T lymphocyte infiltration, CXCR3+ T lymphocytes were required for the chemotaxis and spheroid infiltration assays. Because of the difficulties in detecting CXCR3 on most of the T lymphocytes freshly isolated from PBMCs of normal donors (Figure S2), CD3/CD28 Dynabeads were used to activate the T lymphocytes and induce the expression of CXCR3. After activation, over 90% of CD3/CD28 Dynabeads treated T lymphocytes were CXCR3+ (Figure S2). Chemotaxis assays revealed that CXCL10 recruited the primed T lymphocytes in a dose-dependent manner (Figure 2B).

In addition, to further confirm whether CXCL10 facilitates T lymphocyte infiltration in GC, GC spheroids were established using NCI-N87 cells transfected with CXCL10 or control plasmid (Figures S3, S4). Compared with the control vector-transfected spheroids, the CXCL10-overexpressing GC spheroids showed significantly high infiltration of T lymphocytes (Figures 2D–F).

### Autophagy Was Activated in GC as Determined by GEPIA Analysis

**Figure 3:**
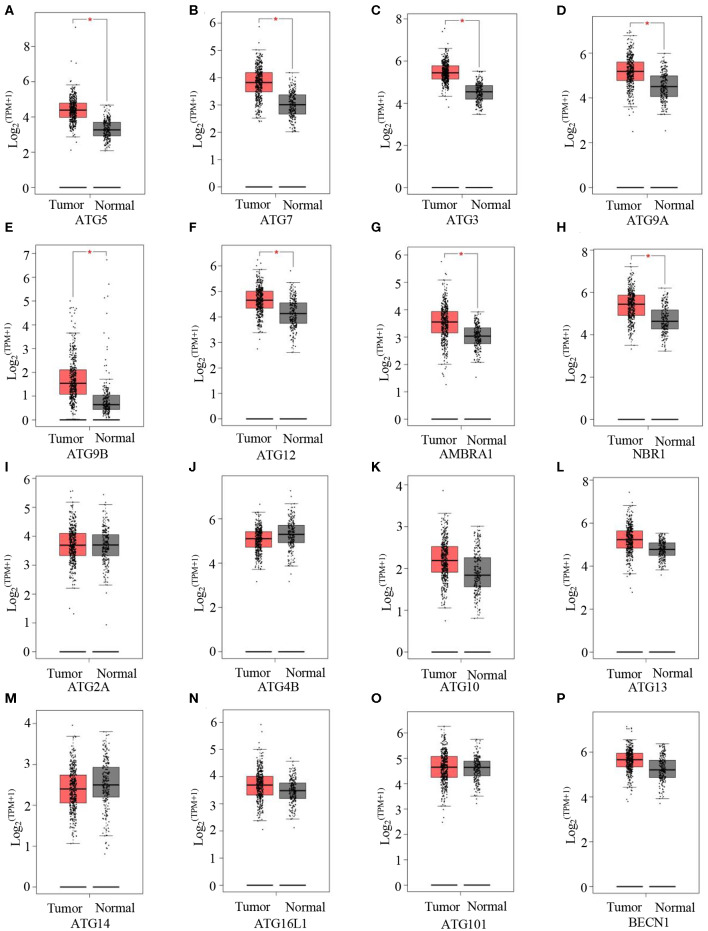
Autophagy is activated in GC. **(A–P)** GEPIA analysis of the expression of ATG5 **(A)**, ATG7 **(B)**, ATG3 **(C)**, ATG9A **(D)**, ATG9B **(E)**, ATG12 **(F)**, ARBRA1 **(G)**, NBR1 **(H)**, ATG2A **(I)**, ATG4B **(J)**, ATG10 **(K)**, ATG13 **(L)**, ATG14 **(M)**, ATG16L1 **(N)**, ATG101 **(O)**, and BECN1 **(P)** in gastric tumors and normal tissues. *P < 0.05.

Next, we evaluated autophagy activation in GC. Here, GEPIA was used to detect the expression levels of a few ATGs between GCs and normal tissues. Compared with normal tissues, tumor tissues showed significantly higher mRNA levels of the following key autophagy genes: ATG5 (Figure 3A), ATG7 (Figure 3B), ATG3 (Figure 3C), ATG9A (Figure 3D), ATG9B (Figure 3E), ATG12 (Figure 3F), AMBRA1 (Figure 3G), and NBR1 (Figure 3H). These data indicate increased autophagy in GCs.

### Autophagy Inhibition Enhanced CXCL10 Expression in AGS Cells

**Figure 4:**
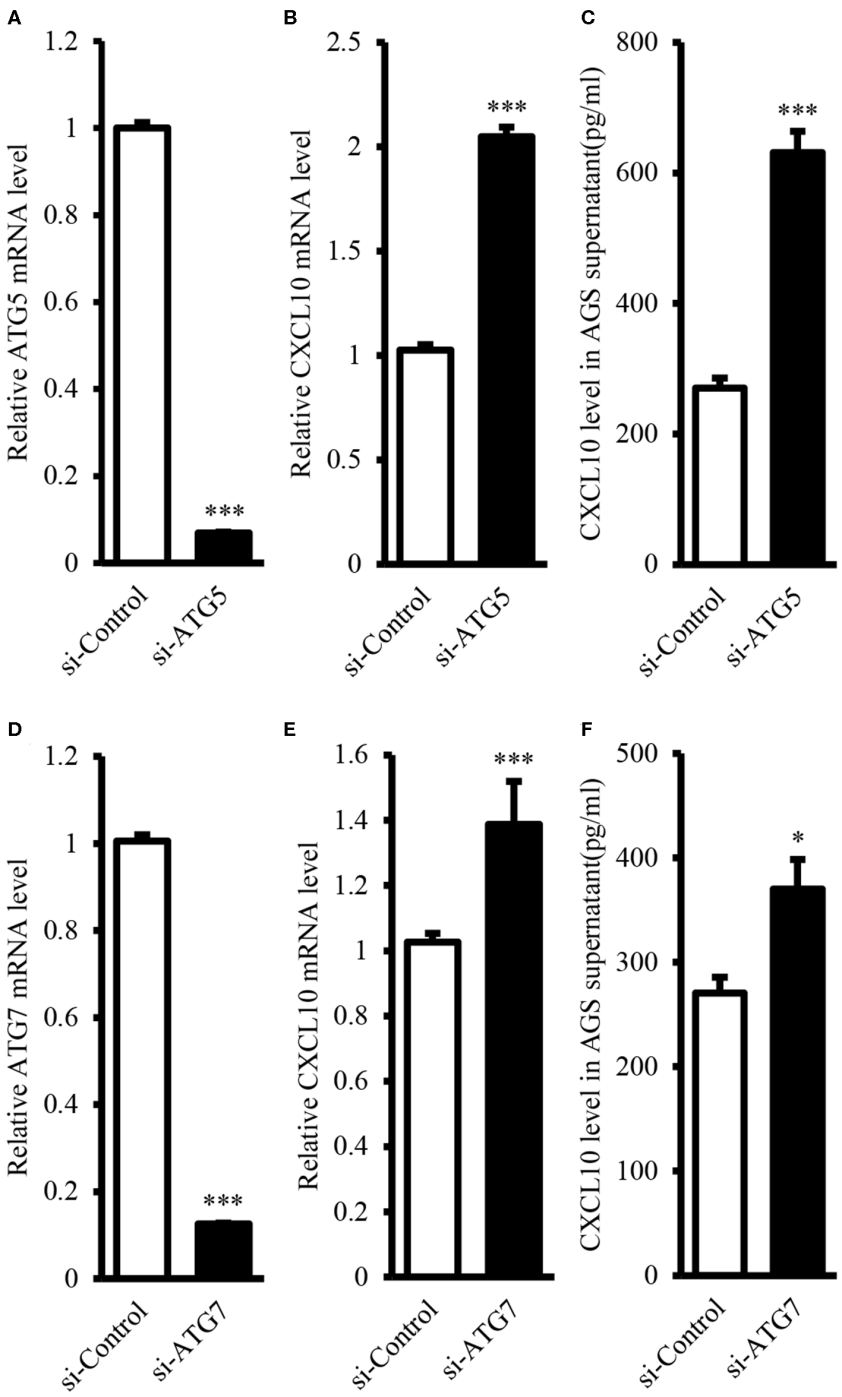
ATG5 and ATG7 knockdown induced CXCL10 expression in AGS cells. **(A)** mRNA expression level of ATG5 in AGS cells transfected with ATG5 siRNA and control siRNA. **(B)** mRNA expression level of CXCL10 in AGS cells transfected with ATG5 siRNA and control siRNA. **(C)** CXCL10 protein level in the culture supernatant of AGS cells transfected with ATG5 siRNA and control siRNA. **(D)** mRNA expression level of ATG7 in AGS cells transfected with ATG7 siRNA and control siRNA. **(E)** mRNA expression level of CXCL10 in AGS cells transfected with ATG7 siRNA and control siRNA. **(F)** CXCL10 protein level in the culture supernatant of AGS cells transfected with ATG7 siRNA and control siRNA. *P < 0.05, ***P < 0.001. Data represent mean ± SE.

It is well-known that ATG proteins are critical for the formation of autophagosome and the activity of autophagy (44, 45). ATG5 and ATG7 are two of the most important components of the ATG family; therefore, ATG5 or ATG7 ablation is sufficient to impair autophagic functions (46–52). In this study, we aimed to induce ablation of ATG5 or ATG7 in AGS cells, as AGS cells showed the highest endogenous CXCL10 expression level among the available GC cell lines (Figure S3). ATG5 siRNA transfection in AGS cells significantly suppressed ATG5 expression at both mRNA (Figure 4A) and protein levels (**Figures 7A,F**). Such ATG5 knockdown inhibited autophagy, as demonstrated by decreased LC3II/LC3I ratio (**Figures 7A,E**). In addition, ATG5 knockdown significantly induced CXCL10 mRNA expression in AGS cells (Figure 4B) and significantly increased CXCL10 secretion by AGS cells (Figure 4C). Similarly, ATG7 knockdown significantly induced CXCL10 expression at both mRNA and protein levels (Figures 4E,F).

**Figure 5:**
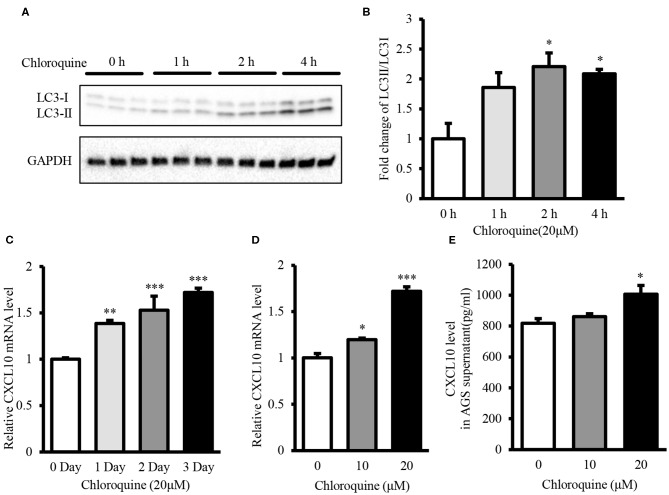
CQ treatment induced CXCL10 expression in AGS cells. **(A)** Western blots showing LC3 expression in 20 μM CQ treated AGS cells. **(B)** Fold change of LC3II/LC3I ratio in 20 μM CQ treated AGS cells. **(C)** Time dependent CXCL10 mRNA expression in 20 μM CQ treated AGS cells. **(D)** CXCL10 mRNA expression in AGS cells treated with different doses of CQ for 3 days. **(E)** Protein level of CXCL10 in the culture supernatant of AGS cells treated with different doses of CQ. *P < 0.05, **P < 0.01, ***P < 0.001. Data represent mean ± SE.

CQ inhibits autophagic flux by decreasing the fusion of autophagosome-lysosome (53). Therefore, we used CQ to further confirm whether autophagy inhibition could induce CXCL10 expression in AGS cells. Treatment with 20 μM CQ significantly induced the accumulation of LC3-II in a time-dependent manner (Figures 5A,B), as reported previously (53–55). Furthermore, 20 μM CQ significantly induced CXCL10 mRNA expression in a time-dependent manner in AGS cells without affecting the cellular viability (Figure 5C, Figure S5). The maximal induction effect was observed at day 3. When incubation time was fixed for 3 days, Treatment with 10 and 20 μM CQ significantly induced CXCL10 mRNA in AGS cells (Figure 5D). In addition, CXCL10 secretion by AGS cells treated with 20 μM CQ was significantly higher than that by control cells (Figure 5E).

### Autophagy Inhibition Facilitated T lymphocyte Migration by Inducing CXCL10 Secretion

**Figure 6:**
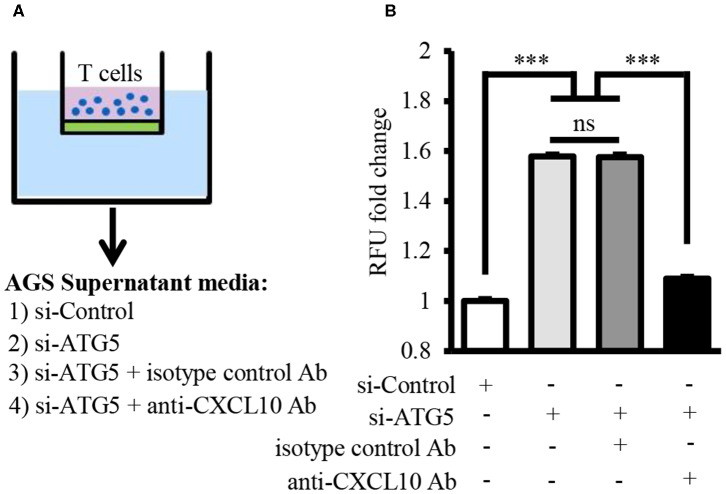
Autophagy inhibition facilitated T lymphocyte migration by inducing CXCL10 secretion. **(A)** Schematic representation of chemotaxis assay for T lymphocyte migration through polycarbonate membrane toward three different mediums. **(B)** Fold change of migrated T lymphocytes. ***P < 0.001. Data represent mean ± SE.

Chemotaxis assay revealed that T lymphocyte recruitment by culture supernatant of ATG5-knockdown AGS cells was significantly higher than that by culture supernatant of control cells (Figures 6A,B). This T lymphocyte recruitment was effectively blocked in the presence of neutralizing anti-CXCL10 antibody (Figure 6B).

### Autophagy Inhibition Enhanced CXCL10 Expression by Suppressing the Inhibitory Effect of JNK Signaling

**Figure 7:**
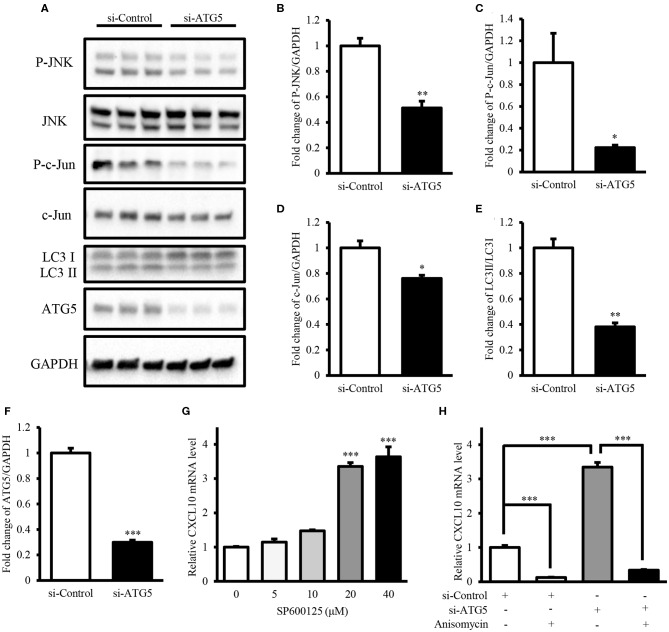
Autophagy inhibition induced CXCL10 expression by suppressing the inhibitory effects of JNK signaling. **(A)** Western blots for phospho-JNK, JNK, phospho-c-Jun, c-Jun, LC3, ATG5, and GAPDH levels in AGS cells transfected with ATG5 siRNA or control siRNA. **(B–F)** Relative protein levels of P-JNK **(B)**, P-c-Jun **(C)**, c-Jun **(D)**, LC3II/LC3I **(E)**, and ATG5 **(F)**. **(G)** CXCL10 mRNA levels in AGS cells treated with different doses of SP600125. **(H)** CXCL10 mRNA levels in ATG5 siRNA transfected AGS cells treated with or without 100 ng/ml anisomycin. *P < 0.05, **P < 0.01, ***P < 0.001. Data represent mean ± SE.

Next, we investigated the mechanism underlying the induction of CXCL10 expression via autophagy inhibition. Here, we demonstrated that ATG5 knockdown was sufficient to inhibit autophagy (Figures 7A,E,F) and investigated the levels of components of the JNK signaling pathway in AGS cells. ATG5 knockdown significantly decreased the levels of phospho-JNK (Figures 7A,B), phospho-c-Jun (Figures 7A,C), and c-Jun (Figures 7A,D), thereby suppressing JNK signaling. Treatment with the JNK inhibitor SP600125 resulted in a dose-dependent increase in CXCL10 mRNA expression in AGS cells, and 20 and 40 μM SP600125 showed a significant increase in CXCL10 mRNA levels (Figure 7G). In addition, treatment with 100 ng/ml anisomycin, a JNK activator, significantly inhibited CXCL10 mRNA expression in control-vector transfected AGS cells and significantly suppressed the ATG5 knockdown-induced increase in CXCL10 mRNA expression (Figure 7H). Collectively, these data suggest that autophagy inhibition induced CXCL10 expression via suppression of the inhibitory effects of JNK signaling.

### Autophagy Inhibition Induced CXCL10 Expression in CoCl_2_-Treated AGS Cells

**Figure 8:**
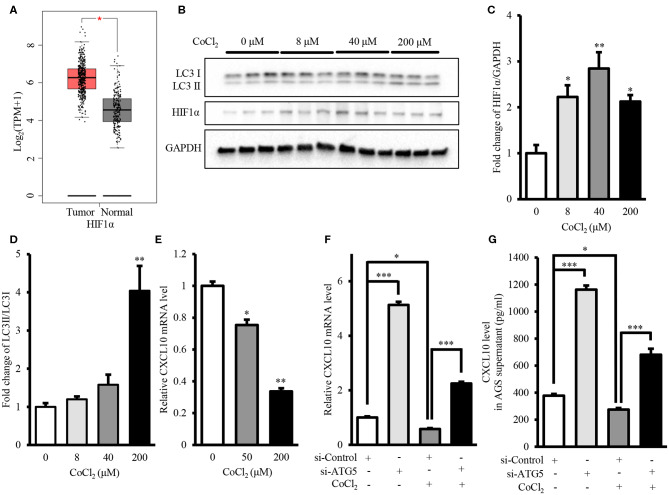
Autophagy inhibition induced CXCL10 expression in CoCl_2_-treated AGS cells. **(A)** GEPIA analysis of HIF1α expression in gastric tumors and normal tissues. **(B)** Western blots for HIF1α and LC3 in AGS cells treated with different concentrations of CoCl_2_. **(C,D)** Relative protein levels of HIF1α **(C)** and LC3II/LC3I ratio **(D)** in AGS cells treated with different concentrations of CoCl_2_. **(E)** CXCL10 mRNA levels of in CoCl_2_-treated AGS cells. **(F)** CXCL10 mRNA levels in ATG5 siRNA transfected AGS cells treated with or without CoCl_2_. **(G)** CXCL10 protein levels in the culture supernatant of ATG5 siRNA transfected AGS cells treated with or without CoCl_2_. *P < 0.05, **P < 0.01, ***P < 0.001. Data represent mean ± SE.

Intra-tumor hypoxia is an important characteristic of 50–60% malignant tumors (56). Moreover, GEPIA showed that mRNA level of HIF1α, the hypoxia marker, in GCs was significantly higher than that in normal gastric tissues (Figure 8A). Therefore, we investigated the effect of autophagy inhibition on CXCL10 expression under hypoxia mimetic conditions. Treatment with CoCl_2_, a hypoxia mimetic reagent, significantly increased HIF1α protein level in AGS cells (Figures 8B,C). Treatment with 200 μM CoCl_2_ significantly increased the LC3II/LC3I ratio, indicating increased autophagic activity in AGS cells (Figures 8B,D). Furthermore, CoCl_2_ decreased CXCL10 expression in a dose-dependent manner, and both 50 and 200 μM CoCl_2_ significantly decreased CXCL10 mRNA levels in AGS cells (Figure 8E). ATG5 knockdown significantly increased CXCL10 expression in CoCl_2_ treated AGS cells at both mRNA and protein levels (Figures 8F,G).

## Discussion

**Figure 9:**
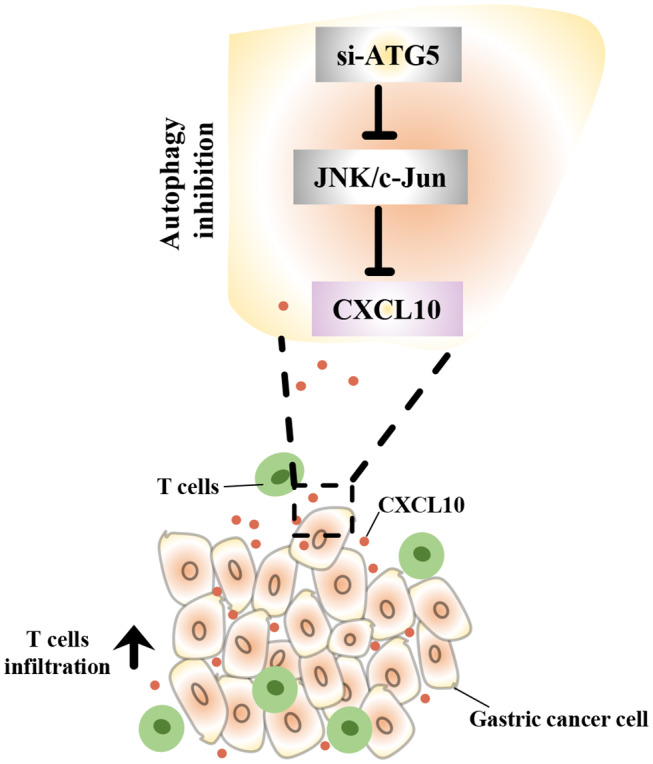
Schematic representation of the mechanism underlying the increased CXCL10 expression in autophagy inhibited GC cells. In GC cells, autophagy inhibition suppressed JNK signaling and subsequently induced CXCL10 expression. As a result, increased CXCL10 recruited more T lymphocytes into gastric tumors.

In this study, we demonstrated that intra-tumor CXCL10 is an important chemokine that contributes to intra-tumor infiltration of T lymphocytes in GC. We also showed that autophagy inhibition could effectively facilitate T lymphocyte migration into the tumor microenvironment by inhibiting the JNK pathway and further inducing the expression of CXCL10 (Figure 9). This might represent a novel therapeutic strategy to enhance the effectiveness of solid tumor immunotherapies such as immune check-point blockade.

It is well-known that the levels of T lymphocyte infiltration into the tumor determine the efficacy of immunotherapy. Primed T lymphocytes gain the expression of certain homing molecules (such as CXCR3) on their surface and thus obtain the capability to migrate toward the tumor site (24). In our study, CXCL10, the well-accepted CXCR3 ligand, functioned as a chemoattractant for T lymphocytes (Figures 2A,B) and recruited T lymphocytes to GC spheroids (Figures 2C–F). Moreover, CXCL10 expression was positively correlated with overall survival (Figure 1A) and relapse-free survival (Figure 1B) in patients with GC. Consistent with our observations, Barash et al. indicated that CXCL10 administration not only induced the infiltration of T cells and NK cells into myeloma tumors but also reduced the accumulation of Treg cells at the tumor site, thereby suppressing tumor progression (57). In addition, CD26 inhibition was reported to enhance T lymphocyte trafficking into melanoma tumor by inducing the intra-tumor expression of CXCL10, further improving the efficacy of immunotherapy (58). In addition to being a potent chemoattractant for T lymphocytes, CXCL10 also inhibits tumor growth via suppressing angiogenesis (59–63). Furthermore, CXCL10 overexpression improved the radiosensitivity of tumors in a rodent cervical cancer model (64). In total, the evidence suggests that CXCL10 could be a potential novel candidate for the GC targeted therapy.

Considering the fact that autophagy was not measurable, the indicators for autophagy activation were judged by expression of ATGs. In our study, GEPIA indicated that the expression of some key autophagy genes in GC were significantly higher than that in normal tissue (Figure 3). These results were consistent with previous observations in established solid tumors (32, 65). However, previous findings on the regulatory effect of autophagy inhibition on CXCL10 expression are not consistent. For instance, two studies showed that ATG5 knockdown significantly suppressed influenza-virus induced CXCL10 expression in macrophages (66, 67). Two other studies reported that deletion of some other key autophagy genes, FIP200 or BECN1, led to increased CXCL10 production in mammary tumor cells (68) or melanoma cells (36). Nevertheless, the regulation of CXCL10 expression in GC cells has not yet been reported.

Data from our study showed that autophagy inhibition induced CXCL10 expression in AGS cells. Autophagy inhibition was achieved by two approaches: genetic approach (ATG5 knockdown or ATG7 knockdown) and chemical treatment (CQ). Of note, ATGs is critical for the formation of autophagosome. Autophagy deficiency has been confirmed in cells lacking ATG3 (69), ATG5 (70), BECN1 (71), ATG7 (52), ATG9A (72), ATG16L1 (73), FIP200 (74), and AMBRA1 (75). In addition, CQ, a widely used autophagy inhibitor, is known to inhibit autolysosome formation and lysosomal protein degradation (76). In our study, both genetic approach (ATG5 knockdown or ATG7 knockdown) and chemical treatment (CQ) significantly induced CXCL10 expression in AGS cells, but the mechanism for induction of CXC10 expression was still unclear. Furthermore, our data showed that ATG5 knockdown facilitated T lymphocyte migration by increasing CXCL10 expression.

We next investigated the mechanism underlying the induction of CXCL10 expression by autophagy inhibition. We found that JNK activator decreased and JNK inhibitor increased CXCL10 expression in AGS cells. In addition, autophagy inhibition significantly decreased the activity of JNK signaling pathway. Thus, these data suggest that autophagy inhibition induces CXCL10 expression by suppressing the inhibitory effect of JNK signaling in AGS cells. In contrast, Mgrditchian et al. reported that BECN1 deletion induced CCL5 expression by activating the JNK signaling pathway, which in turn recruited more NK cells into melanoma tumors (36). This difference in the effect of autophagy inhibition on JNK signaling may be associated with tumor types.

Next, we investigated whether autophagy inhibition also induced CXCL10 expression under hypoxia mimetic conditions. Because of the inadequate oxygen supply and increased energy consumption within the tumor microenvironment, hypoxia is one of the most important characteristics of solid tumors, especially in the advanced stages (77). In the hypoxic microenvironment, autophagy flux is enhanced along with increased tumor growth (78). Advanced tumors have been shown to use autophagy to promote tumor survival (79, 80). Our current observations that ATG5 knockdown induced CXCL10 expression in CoCl_2_-treated AGS cells support a scientific basis of autophagy inhibition as a potential combinational therapy strategy for immunotherapy.

Apart from recruiting T lymphocytes into solid tumors and enhancing the sensitivity to anti-tumor therapy, autophagy deficiency was also reported to cause some cancer related pathology (81, 82). For instance, the mutation of ATGs was reported in tumor cells (83). Because of the function of autophagy in counteracting cellular stress, some ATGs were considered as tumor suppressors in rodent tumor models (45, 84–86). In addition, Yang et al. indicated that fluorouracil inhibited the growth of GC cells via ATG6 activation (87). In this case, autophagy also sometimes seems as a protective mechanism in tumor initiation period. Overall, autophagy might regulate tumorigenesis in a tumor stage-specific manner.

In summary, to the best of our knowledge, this is the first report on the regulatory effects of *in vitro* autophagy inhibition on CXCL10 expression in GC cells and its potential mechanism in recruiting T lymphocytes into the tumor. These findings provide novel insights into understanding the functions of autophagy in immunotherapy. Furthermore, our results highlight the potential of autophagy inhibition to be used in combination with immunotherapy approaches such as immune checkpoint blockade. Our findings also suggest CXCL10 as a potential novel candidate for targeted therapy against GC.

## Data Availability Statement

Publicly available datasets were analyzed in this study. Kaplan Meier-plotter can be found here: http://kmplot.com/analysis/. The GEPIA (Gene Expression Profiling Interactive Analysis) can be found here: http://gepia.cancer-pku.cn/index.html.

## Author Contributions

QM and LH designed the study. QM and YZ performed the experiments and statistical analysis. QM drafted the manuscript. QM, YZ, and LH revised the manuscript. All authors read and approved the final manuscript.

## Conflict of Interest

All authors were employed by the company Amgen Biopharmaceutical R&D (Shanghai) Co., Ltd.
