# Supplementary material for: Targeting Autophagy Facilitates T Lymphocyte Migration by Inducing the Expression of CXCL10 in Gastric Cancer Cell Lines
Source: Front Oncol. 2020 Jun 2;10:886. doi: 10.3389/fonc.2020.00886 (PMC7280490; doi:10.3389/fonc.2020.00886)
Supplement: Supplementary file 1 [file Data_Sheet_1.docx]

Supplementary Figure 1

**
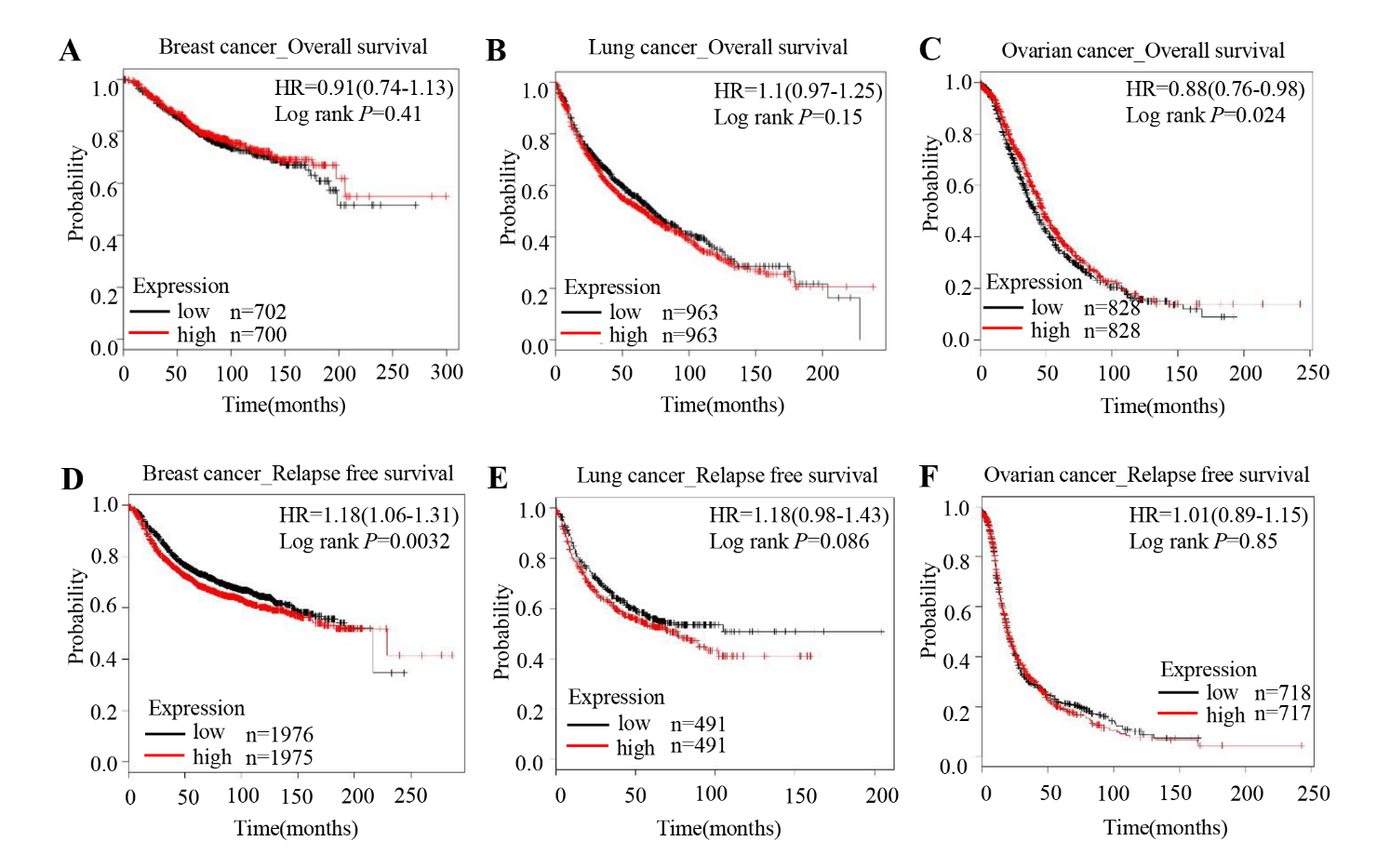
**

**Figure S1** | Correlation between CXCL10 expression and overall survival and relapse-free survival in patients with breast cancer, lung cancer, and ovarian cancer. (A) Kaplan-Meier analysis of overall survival in patients with breast cancer with high CXCL10 expression and low CXCL10 expression (*P* = 0.41, n = 702 and 700 respectively). (B) Kaplan-Meier analysis of overall survival in patients with lung cancer with high-CXCL10 expression and low-CXCL10 expression (*P* = 0.15, n = 963). (C) Kaplan-Meier analysis of overall survival in patients with ovarian cancer with high-CXCL10 expression and low-CXCL10 expression (*P* = 0.024, n = 828). (D) Kaplan-Meier analysis of relapse free survival in patients with breast cancer with high-CXCL10 expression and low-CXCL10 expression (*P* = 0.0032, n = 1976 and 1975 respectively). (E) Kaplan-Meier analysis of relapse free survival in patients with lung cancer with high-CXCL10 expression and low-CXCL10 expression (*P* = 0.086, n = 491). (F) Kaplan-Meier analysis of relapse free survival in patients with ovarian cancer with high-CXCL10 expression and low-CXCL10 expression (*P* = 0.85, n = 718 and 717 respectively)

**Supplementary Figure 2**

**
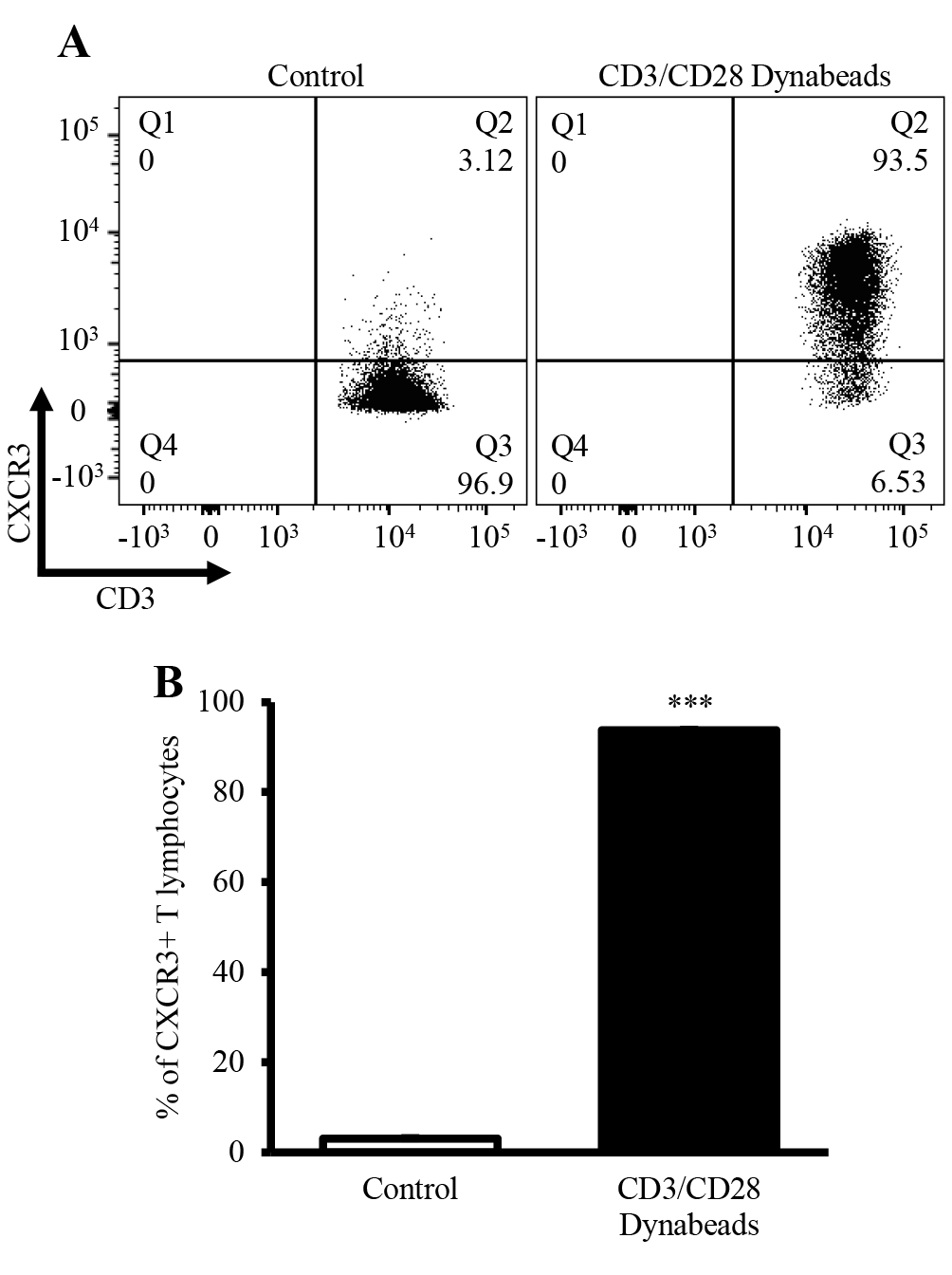
**

**Figure S2** | CD3/CD28 Dynabeads treatment induced CXCR3 expression in CD3+ T lymphocytes. (A) Representative flow cytometry analysis demonstrating higher proportion of CXCR3+ T lymphocytes among CD3/CD28 Dynabeads-treated CD3+ T lymphocytes. (B) Proportion of CXCR3+ T lymphocytes in CD3+ T lymphocytes. ^***^*P* < 0.001. Data represent mean ± SE.

**Supplementary Figure 3**

**
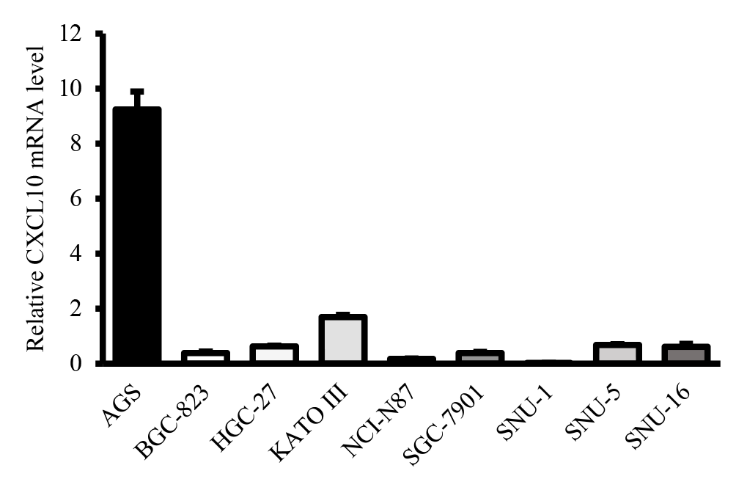
**

**Figure S3** | CXCL10 expression was the highest in AGS cells, compared with that in other eight gastric cancer cell lines. During data analysis, ΔCt (control group) = 12 was used for normalization. Data represent mean ± SE.

**Supplementary Figure 4**

**
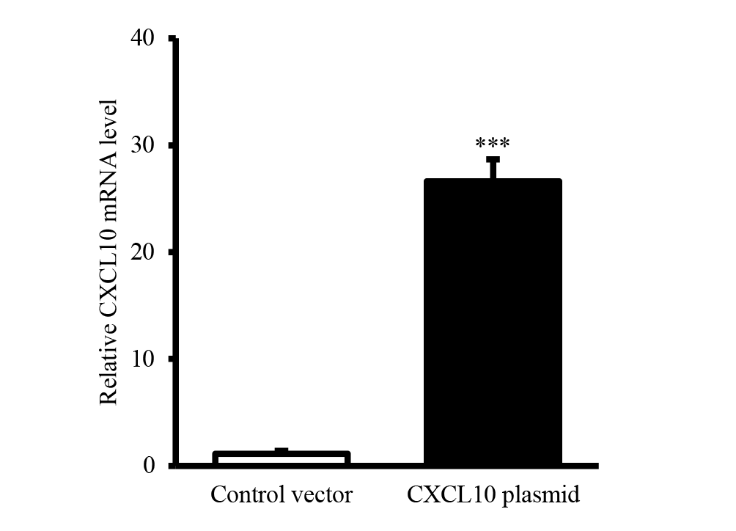
**

**Figure S4** | CXCL10 plasmid transfection induced CXCL10 overexpression in NCI-N87 cells. CXCL10 mRNA level in CXCL10 plasmid-transfected NCI-N87 cells was significantly higher than that in control vector-transfected NCI-N87 cells at 48 hours after transfection. ^***^*P* < 0.001. Data represent mean ± SE.

**Supplementary Figure 5**

**
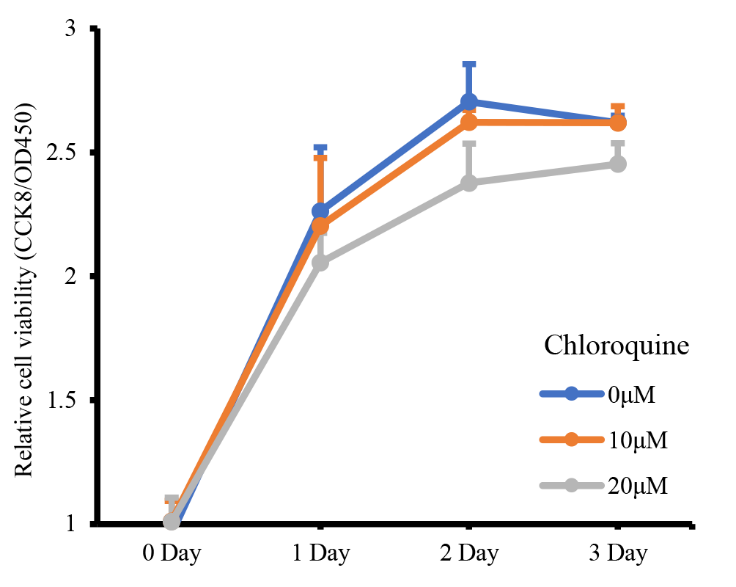
**

**Figure S5** | Treatment with 10µM and 20µM CQ did not significantly affect the viability of AGS cells at the following time points: 1 day, 2 day and 3 day. Data represent mean ± SE.
